# Supplementary material for: Epigenetic modulation of Fgf21 in the perinatal mouse liver ameliorates diet-induced obesity in adulthood
Source: Nat Commun. 2018 Feb 12;9:636. doi: 10.1038/s41467-018-03038-w (PMC5809372; doi:10.1038/s41467-018-03038-w)
Supplement: Supplementary file 1 — Supplementary Information [file 41467_2018_3038_MOESM1_ESM.pdf]

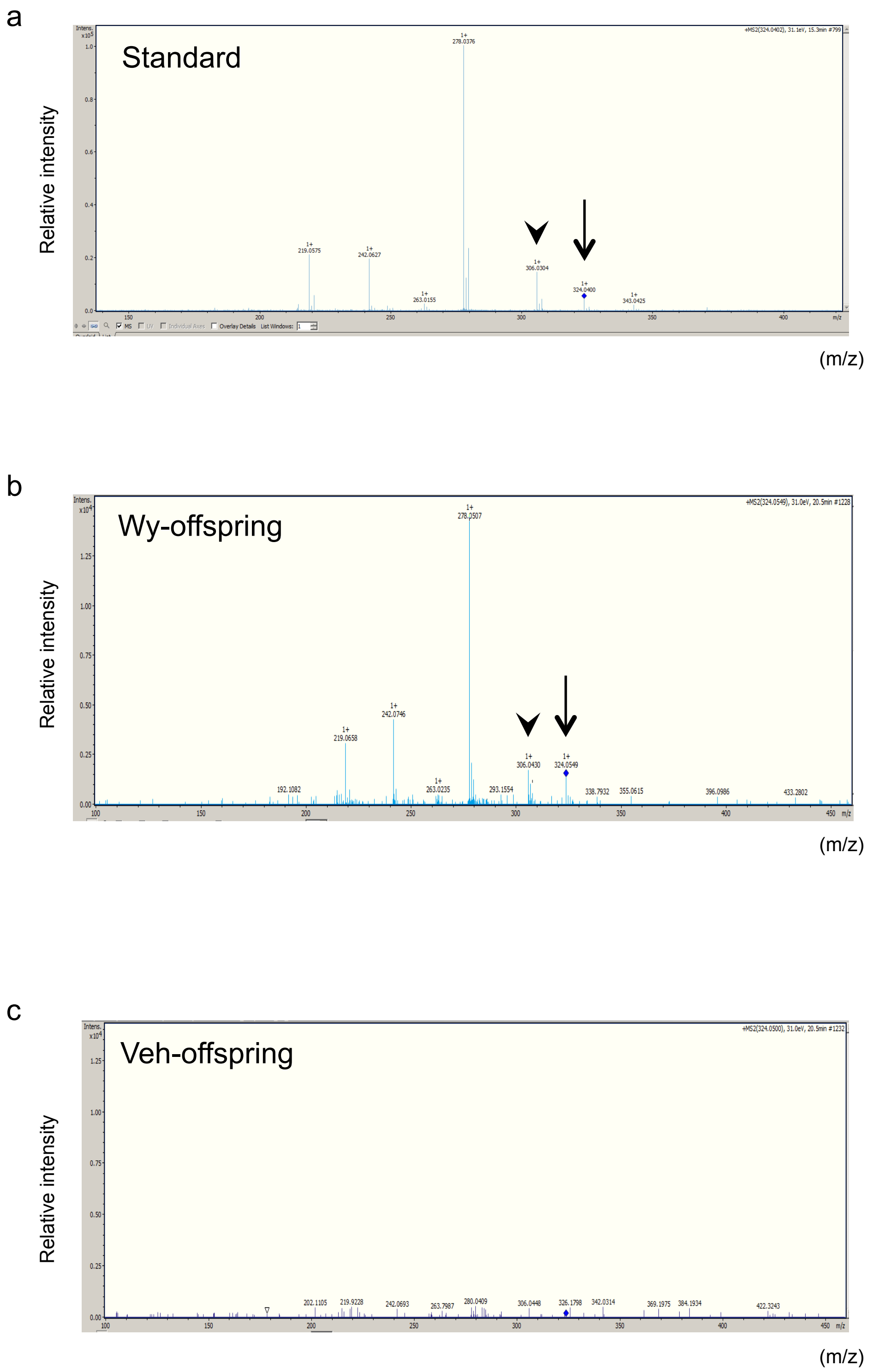

Supplementary Fig. 1 Analysis of the gastric contents of pups at D16 by LC/MS-MS
a Standard (0.1μg/ml Wy dissolved in 50% acetonitrile). **b, c** The gastric contents of Wy- (**b**) and Veh- (**c**) offspring. Arrow and arrowhead indicate precursor and product peak specific to Wy, respectively. m/z: mass-to-charge ratio.

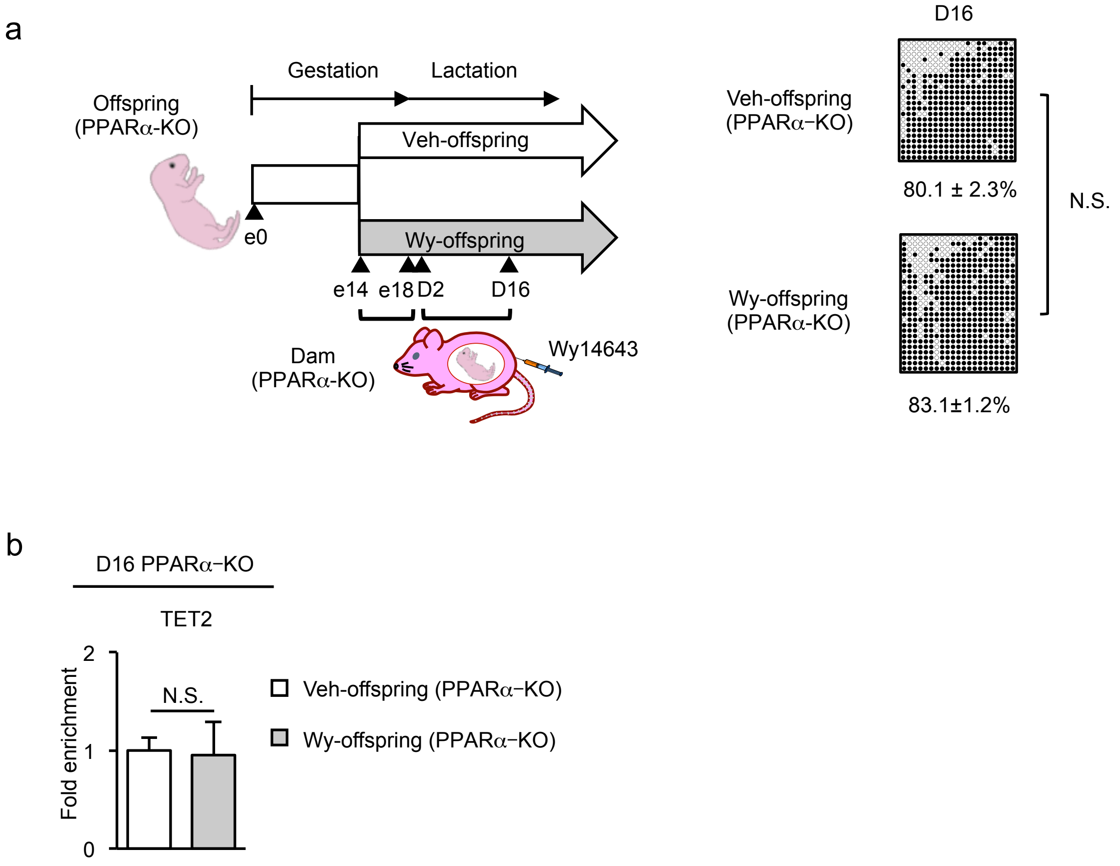

Supplementary Fig. 2 DNA methylation analysis of *Fgf21* in PPARα-KO offspring

a Bisulfite sequencing analysis of *Fgf21* in offspring derived from Wy- and DMSO (Veh)-treated PPARα-KO dams at D16. (n = 4–5 per group). **b** ChIP assays for TET2 at D16 in PPARα-KO mice. (n = 3 per group). Statistics by unpaired Student's t-test. Data are expressed as mean ± SEM. N.S., not significant.

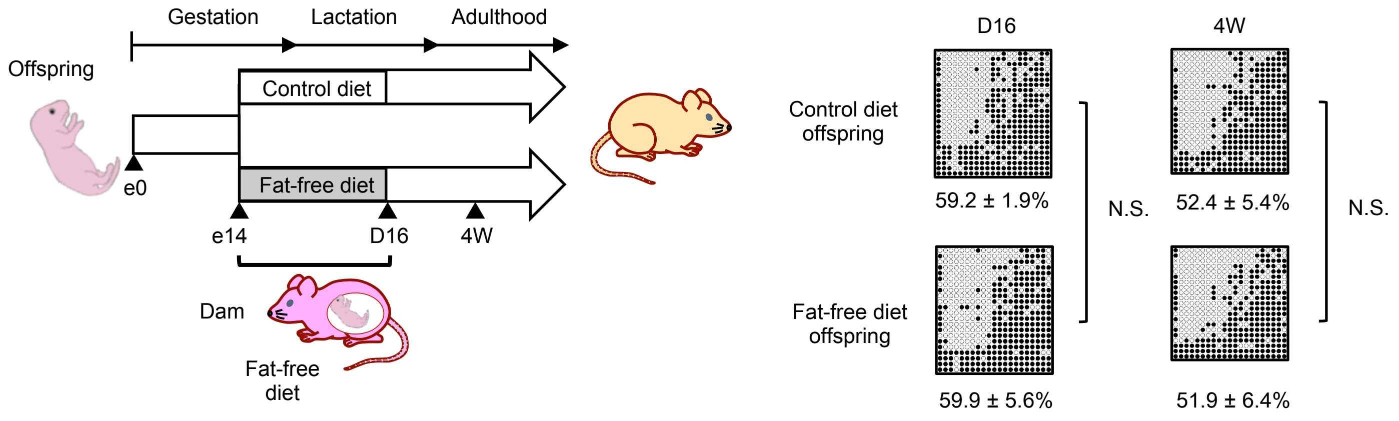

Supplementary Fig. 3 DNA methylation analysis of *Fgf21* in offspring derived from Fat-free diet fed dams

Experimental protocol (left) and representative bisulfite sequencing analysis of *Fgf21* (right) in offspring derived from Control or Fat-free diet fed dams at D16 and 4W. n = 5 per group. Statistics by unpaired Student's t-test. Data are expressed as mean ± SEM. N.S., not significant.

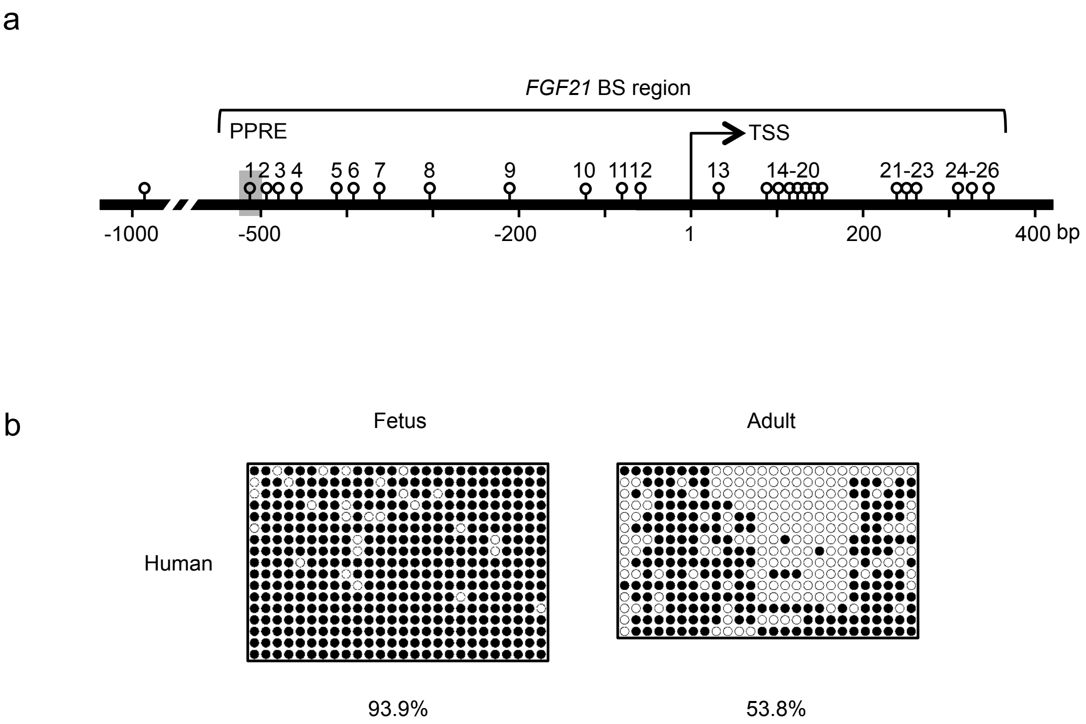

Supplementary Fig. 4 DNA methylation analysis of *FGF21* in fetal and adult human livers
a Schematic representation of the promoter region of *FGF21*. Open circles and the gray box indicate CpG sites and a PPAR response element (PPRE), respectively. Bisulfite sequencing (BS) analysis encompassing the transcription start site (TSS) is indicated. **b** BS analysis of *FGF21* in fetal and adult human livers (representative data of 3 individuals per group).

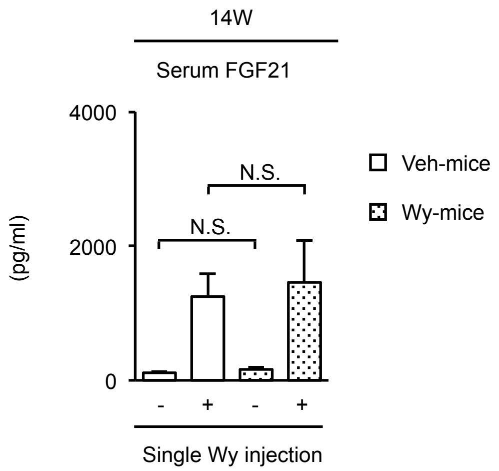

Supplementary Fig. 5 Serum FGF21 concentrations after a single Wy injection in Wy- and Veh-mice at 14W *n* = 4-5 per group. Statistics by unpaired Student's *t*-test. Data are expressed as mean ± SEM. N.S., not significant.

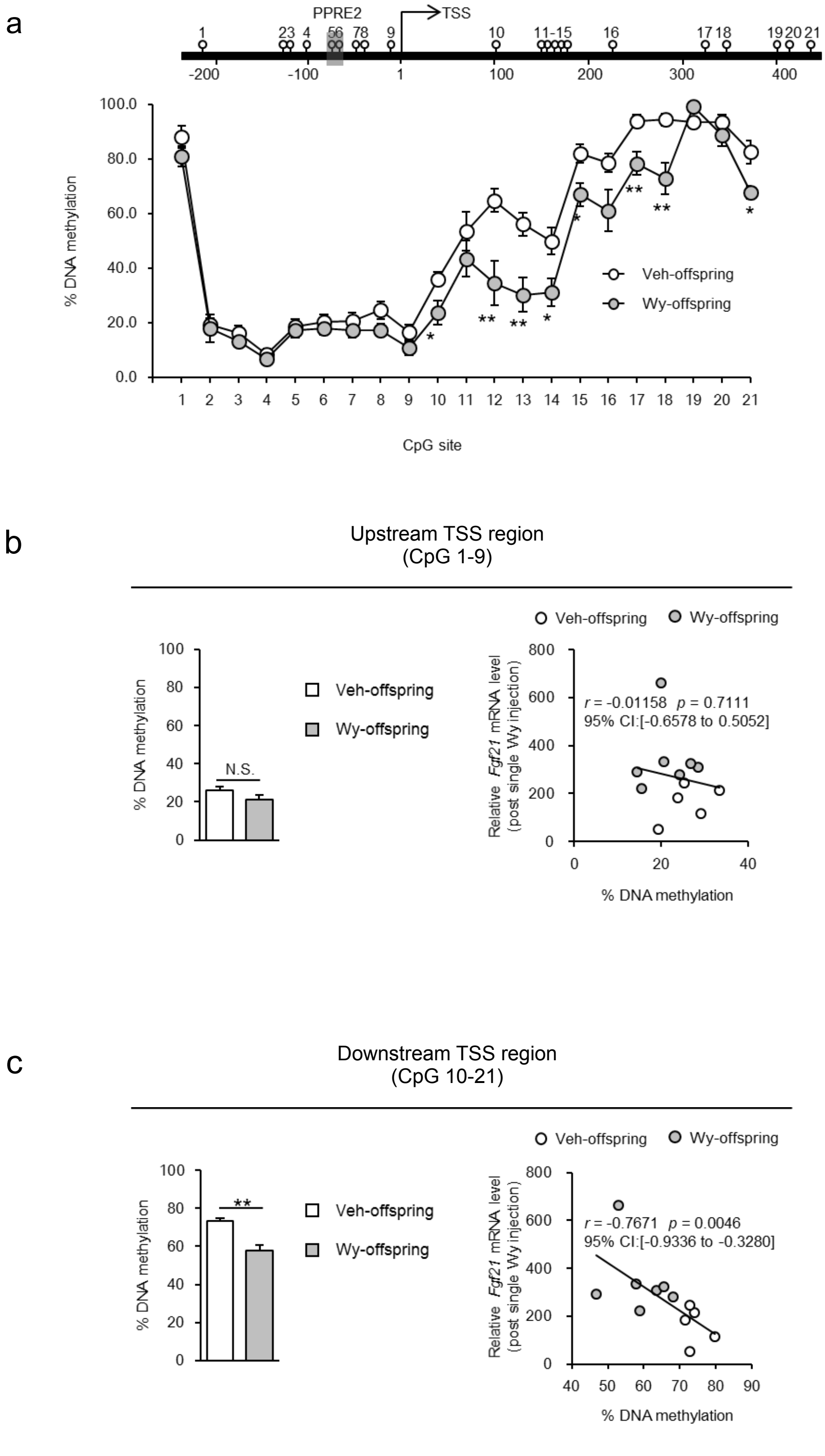

Supplementary Fig. 6 Statistical analysis of DNA methylation ratio at each CpG site of *Fgf21*
a Schematic representation of the TSS flanking region of *Fgf21* (top) and DNA methylation ratio of each CpG site in *Fgf21* (bottom) (n = 5-7 per group). **b, c** DNA methylation ratio (left) of CpG sites located upstream (#1-9) and downstream (#10-21) of the TSS of *Fgf21* (n = 5-7 per group). Correlation between *Fgf21* mRNA expression after a single Wy injection and DNA methylation ratio (right). (n = 12). Statistics by unpaired Student's t-test (**a, b-c** left) or Spearman's rank correlation coefficient (**b-c** right). The r and p values are indicated on the graph. Data are expressed as mean ± SEM. *$P < 0.05$; ** $P < 0.01$; N.S., not significant vs. Veh-offspring.

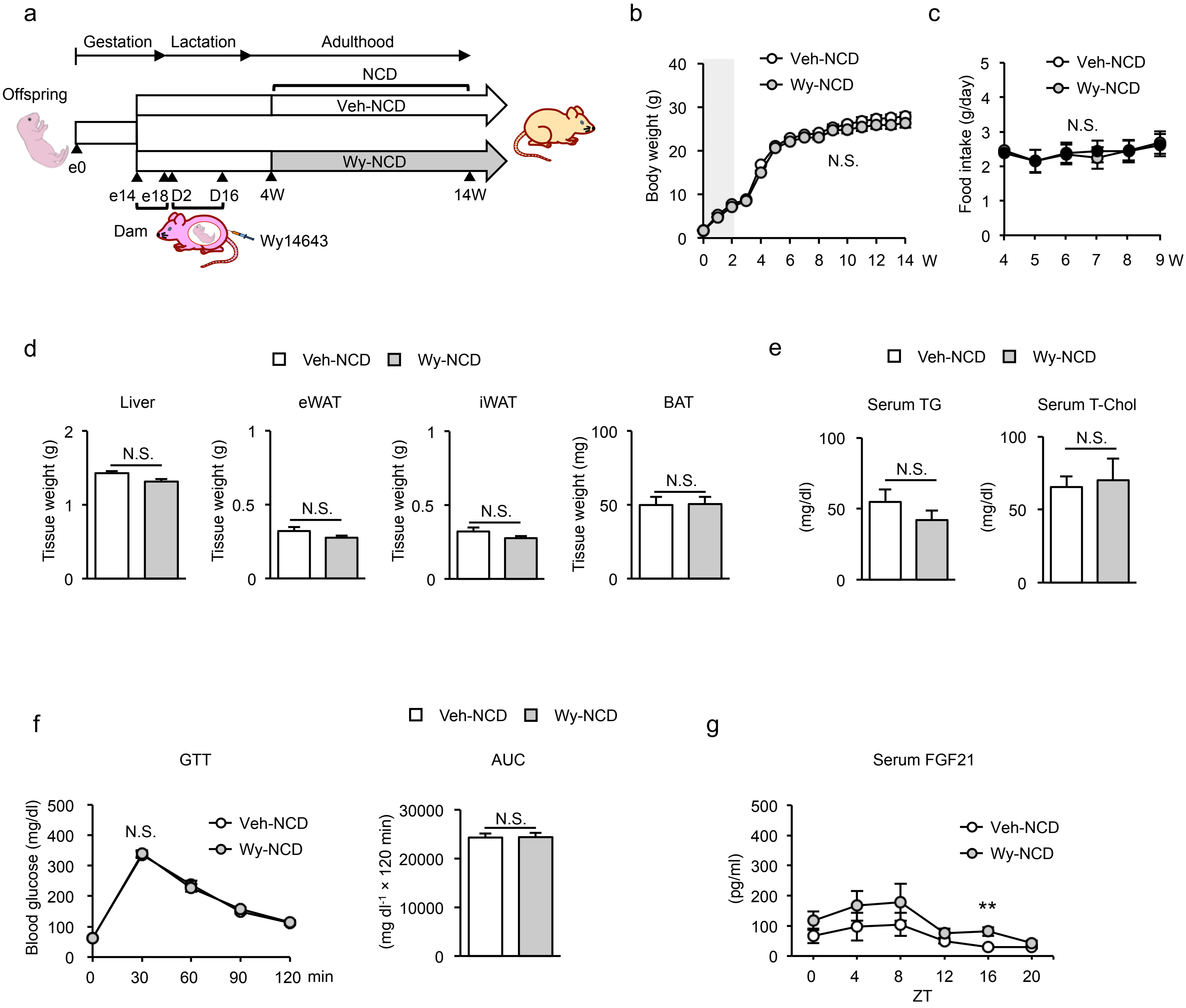

Supplementary Fig. 7 Metabolic phenotypes of Wy-offspring during NCD feeding

a Experimental protocol of Veh-offspring and Wy-offspring fed NCD diet, which are referred to as Veh-NCD and Wy-NCD, respectively. **b, c** Body weight changes (**b**) and food intake (**c**) during NCD feeding. **d** Tissue weights of Veh-NCD and Wy-NCD at 14W. **e** Serum TG and T-Chol levels. **f** Intraperitoneal glucose tolerance test (GTT) (left) and the area under the curve (AUC) (right). **g** Circadian variation of serum FGF21 concentrations. ZT, zeitgeber time. n = 6-10 per group. Statistics by unpaired Student's t-test (**d, e, f, g**) or two-way ANOVA with repeated measures (**b, c**). Data are expressed as mean ± SEM. ** $P < 0.01$; N.S., not significant vs. Veh-NCD.

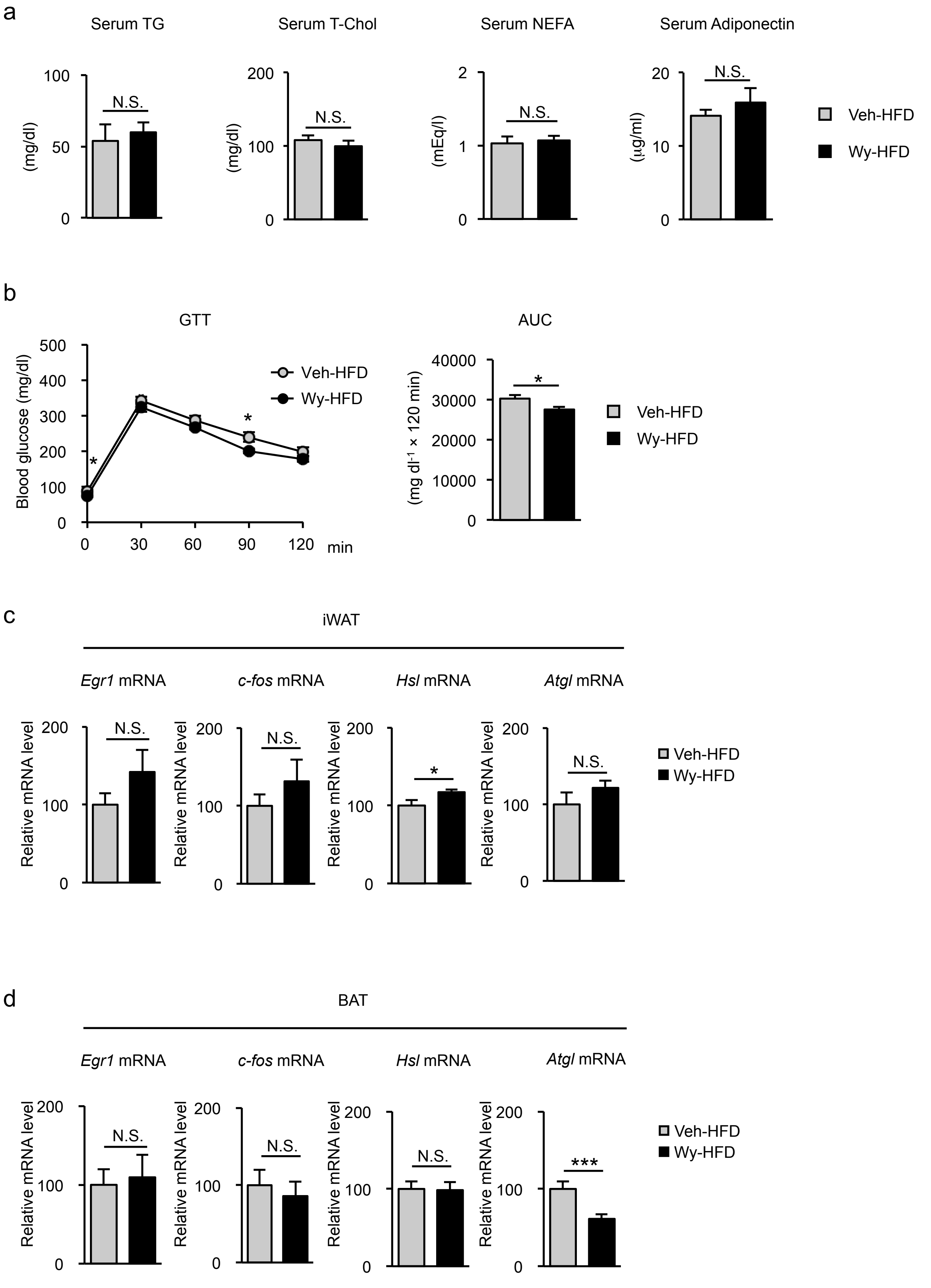

Supplementary Fig. 8 Metabolic phenotypes of Wy-offspring during HFD feeding
a Serum TG, T-Chol, NEFA, and adiponectin concentrations. **b** Glucose tolerance test (GTT) (left) and the area under the curve (AUC) (right). **c, d** Relative mRNA expression of *Egr1*, *c-fos*, *Hsl*, and *Atgl* in iWAT **(c)** and BAT (**d**). n = 10-11 per group. Statistics by unpaired Student's t-test. Data are expressed as mean ± SEM. *P < 0.05; *** P < 0.001; N.S., not significant vs. Veh-HFD.

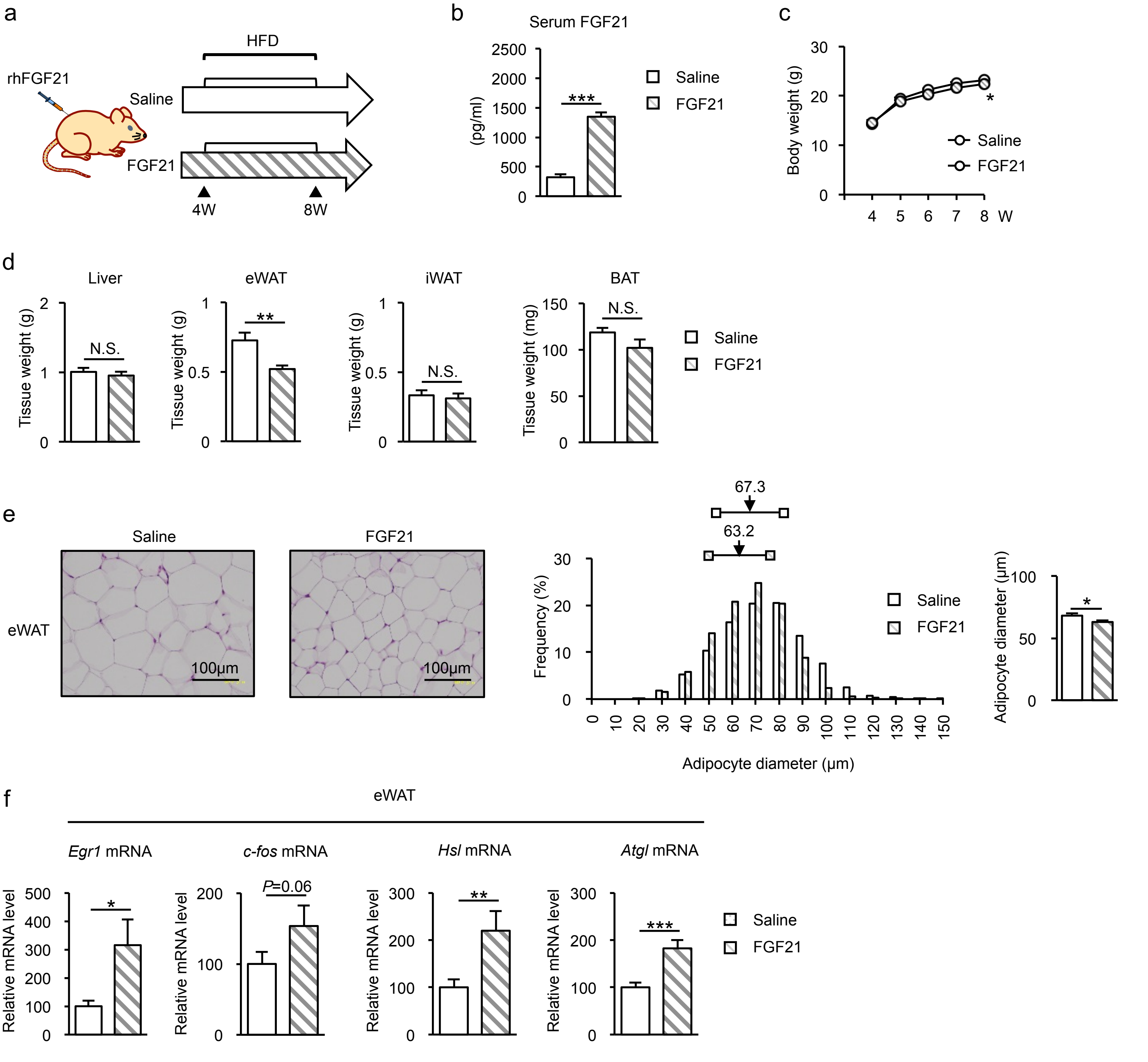

Supplementary Fig. 9 Recombinant human FGF21 administration in adult mice during HFD feeding

a Experimental protocol. **b** Serum FGF21 concentrations in saline- and FGF21-treated HFD-fed mice at 8W. **c** Body weight changes (n = 8 per group; statistics by two-way ANOVA with repeated measures) and **d** tissue weights in FGF21- and saline-treated HFD-fed mice at 8W. **e** Hematoxylin and eosin (HE) staining (left, representative image of 8 individuals per group) and quantification of adipocyte diameter (middle and right) of eWAT. Histograms of adipocyte diameter (middle). Horizontal lines with bilateral squares indicate interquartile range (IQR). Arrows indicate the median values (numbers above the horizontal lines) of saline- and FGF21-treated HFD-fed mice. Statistical analysis (right) of mean adipocyte diameters. Scale bar = 100 μm. **f** Relative mRNA expression of *Egr1*, *c-fos*, *Hsl*, and *Atgl* in eWAT. n = 8 per group. Statistics by unpaired Student's t-test otherwise indicated. Data are expressed as mean ± SEM. *$P < 0.05$; ** $P < 0.01$; *** $P < 0.001$; N.S., not significant vs. saline-treated HFD-fed mice.

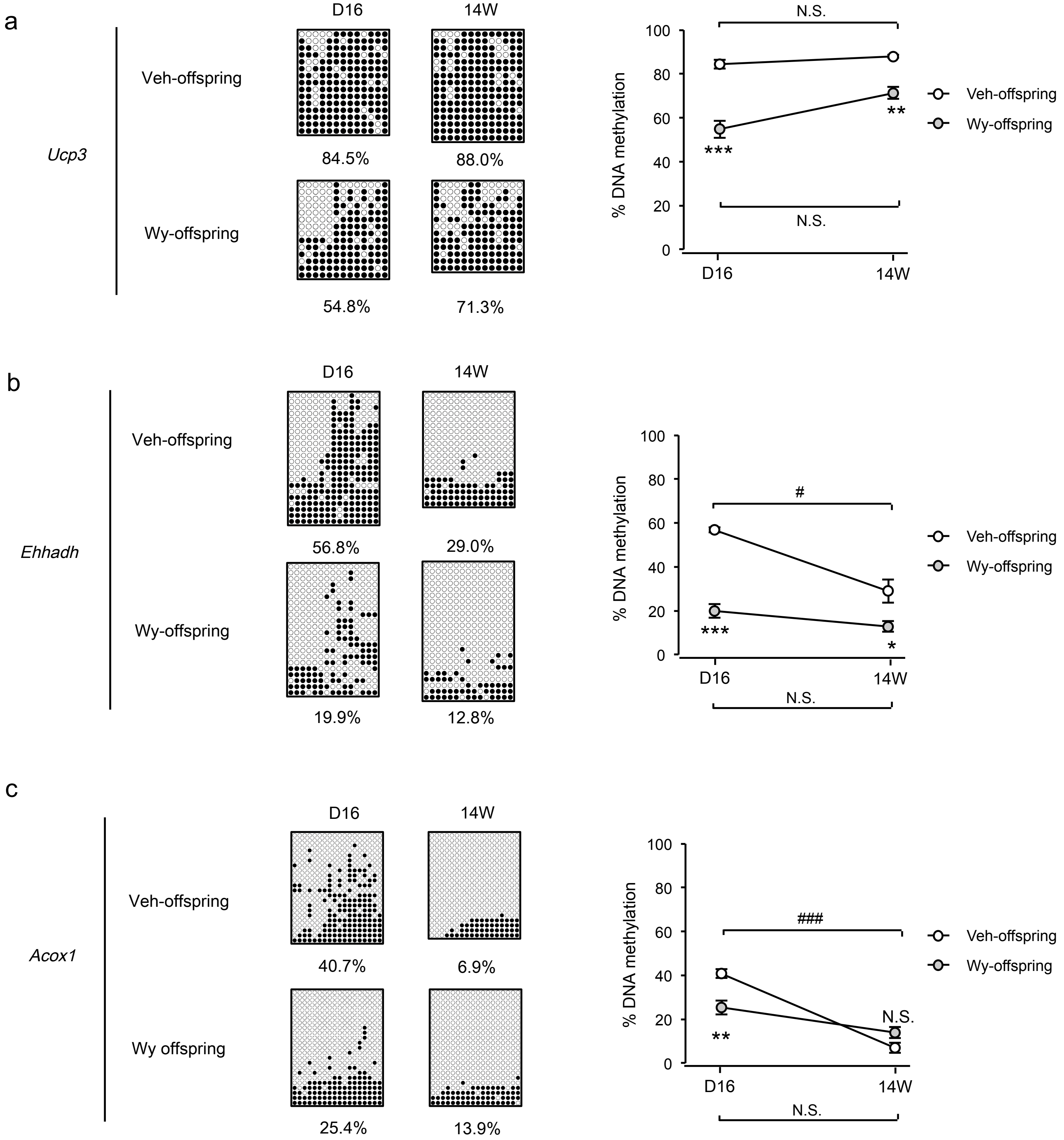

Supplementary Fig. 10 DNA Methylation analysis of other PPARα target genes

Representative bisulfite sequencing analysis (left) and graphic presentation of statistical analysis (right) of the bisulfite sequencing data of *Ucp3* (**a**), *Ehhadh* (**b**) *and Acox1* (**c**) in Wy- and Veh-offspring. n = 3-6 per group. Statistics by unpaired Student's t-test. Data are expressed as mean ± SEM. *$P < 0.05$; ** $P < 0.01$; *** $P < 0.001$; N.S., not significant of Wy-offspring vs. Veh-offspring. $^{\#}P < 0.00$; $^{\#\#\#}P < 0.001$; N.S., not significant of 14W vs. D16.

Supplementary Table 1 Milk fatty acid composition (Male offspring at D16)

		**Veh-offspring (n=4)**	**Wy-offspring (n=4)**	***P*-value**
capric acid	n-10:0	5.14±0.50	6.06±0.48	0.11
lauric acid	n-12:0	9.14±0.95	10.8±0.75	0.06
myristic acid	n-14:0	10.0±1.32	11.2±0.74	0.34
palmitic acid	n-16:0	22.0±0.83	21.4±0.37	0.34
palmitoleic acid	16:1n-7	2.63±0.22	2.57±0.12	0.49
stearic acid	n-18:0	2.68±0.23	2.41±0.05	0.34
oleic acid	18:1n-9	21.5±2.37	18.4±1.20	0.06
	18:1n-7	N/A	N/A	N/A
linoleic acid	18:2n-6	14.2±0.27	14.4±0.45	0.66
alpha-linolenic acid	18:3n-3	0.61±0.03	0.58±0.03	0.34
	20:1	1.86±0.15	1.69±0.08	0.11
	20:3n-9	1.58±0.08	1.63±0.05	0.54
	20:3n-6	0.99±0.09	1.05±0.04	0.43
arachidonic acid (ARA)	20:4n-6	0.89±0.13	0.98±0.12	0.23
eicosapentaenoic acid (EPA)	20:5n-3	0.28±0.01	0.30±0.02	0.29
	n-22:0	N/A	N/A	N/A
	22:1	0.41±0.03	0.37±0.07	0.46
	22:4n-6	0.44±0.06	0.49±0.03	0.23
	22:5n-6	N/A	N/A	N/A
	22:5n-3	0.57±0.03	0.61±0.04	0.17
docosahexaenoic acid (DHA)	22:6n-3	0.84±0.10	0.80±0.05	0.77
	Others	1.82±0.08	1.84±0.16	0.83

N/A: not applicable

Statistics by unpaired Student’s t-test. Data are expressed as mean ± SEM.

Supplementary Table 2 Milk fatty acid composition under fat-free diet feeding (Male offspring at D16)

		**Control** (Fat 10.0 kcal%, n=6)	**Fat-free** (Fat 0 kcal%, n=6)	***P*-value**
capric acid	n-10:0	6.16±0.73	4.09±0.79	***
lauric acid	n-12:0	13.6±0.19	10.6±2.03	**
myristic acid	n-14:0	19.1±0.32	14.5±3.14	**
palmitic acid	n-16:0	30.1±0.35	26.9±1.92	**
palmitoleic acid	16:1n-7	2.57±0.03	6.19±0.59	***
stearic acid	n-18:0	2.44±0.06	2.30±0.12	*
oleic acid	18:1n-9	15.3±0.18	22.9±4.95	**
	18:1n-7	1.81±0.02	6.16±1.14	***
linoleic acid	18:2n-6	5.01±0.06	0.72±0.06	***
alpha-linolenic acid	18:3n-3	0.36±0.01	0.06±0.02	***
	20:1	0.78±0.03	2.63±0.73	***
	20:3n-9	0.41±0.01	0.23±0.02	***
	20:3n-6	0.29±0.01	0.10±0.01	***
arachidonic acid (ARA)	20:4n-6	0.30±0.02	0.24±0.07	0.071
eicosapentaenoic acid (EPA)	20:5n-3	0.03±0.00	n.d	N/A
	n-22:0	0.03±0.00	0.04±0.01	*
	22:1	0.11±0.01	0.28±0.08	***
	22:4n-6	0.13±0.00	0.06±0.01	***
	22:5n-6	0.05±0.00	0.03±0.01	***
	22:5n-3	0.12±0.01	0.01±0.01	**
docosahexaenoic acid (DHA)	22:6n-3	0.12±0.01	0.15±0.02	**
	Others	1.37±0.10	1.76±0.13	***

n.d: not detected

N/A: not applicable

Statistics by unpaired Student's t-test. Data are expressed as mean ± SEM.

P* < 0.05, ** *P* < 0.01, * *P* < 0.001, vs. Control

Supplementary Table 3 Human fetal and adult liver genomic DNAs purchased from BioChain Institute Inc. (Newark, CA, USA)

Human genomic DNA	Sex	Age	BioChain Catalog #	Lot #
fetal liver	male	18 weeks after fertilization	D1244149	A712254
fetal liver	male	22 weeks after fertilization	D1244149-50	A701341
fetal liver	female	20 weeks after fertilization	D1244149	B608368
adult liver	male	24 years old	D1234149	B809103
adult liver	male	41 years old	D1234149	B405012
adult liver	male	59 years old	D4234149	A701175

Supplementary Table 4 Primer sequences for bisulfite sequencing

Gene	Forward	Reverse
m*Fgf21*	TTTAGTTTTTTTTTTTAGATTTAGGAGTGTAGATT	TCCTCCTCTCAACCTCCATAAAAC
h*FGF21*	GATAGATGAGGTTGAGGTTGGTTTA	CAAACTCTAACCCACACTCACTTT
m*Ucp3*	TGAAAGTTTAAAGGAGTTATATTAAAGAGTTTTAG	ACCTATAAAACCAACCCAACTCTCTCC
m*Ehhadh*	GGTTTAAGTTAAATTTGTAGTAGTTTGGTTGG	AAAACAAAATCTAAATAAATCAACAATTCCT
m*Acox1*	AATTGTGGGAGAGGGTGGGTTA	AAACCATATCTCCAACCCCCTTATAT

Supplementary Table 5 Antibodies used in ChIP assay

Antibody	Manufacturer	Dilution rate
anti-trimethyl-histone H3 (Lys4)	Merck Millipore, 07-473	1:400
anti-acetyl-histone H3 (Lys27)	Abcam, ab4729	1:400
anti-trimethyl-histone H3 (Lys27)	Abcam, ab6002	1:400
anti-dimethyl-histone H3 (Lys9)	Cell Signaling Technology, 9753	1:400
anti-ten eleven translocation (TET) 1	Merck Millipore, 09-872	1:200
anti-ten eleven translocation (TET) 2	Abiocode, 1086-6b	1:200
anti-ten eleven translocation (TET) 3	GeneTex, GTX121453	1:200
anti-DNA methyltransferase (Dnmt)3a	Abcam, ab2850	1:200
anti-DNA methyltransferase (Dnmt)3b	Abcam, ab2851	1:200
anti-RNA polymerase (pol) II	Abcam, ab5408	1:400

Supplementary Table 6 Primer sequences for ChIP assay

Gene	Forward	Reverse
m*Fgf21* (-997/-923bp)	AGGCCCGAATGCTAAGC	AGCCCAGCAGGTGGAAGTCT
m*Fgf21* (-106/+21bp)	TGGAATTCAGGTTCCTGCCAA	GAGAAGACACTAAGGCTGTC

Supplementary Table 7 Primer sequences for RT-qPCR

**Gene**	**Forward**	**Reverse**
m*36B4*	GGCCCTGCACTCTCGCTTTC	TGCCAGGACGCGCTTGT
m*Fgf21*	CCTCTAGGTTTCTTTGCCAACAG	AAGCTGCAGGCCTCAGGAT
m*Egr1*	GACGAGTTATCCCAGCCAAA	GGCAGAGGAAGACGATGAAG
m*c-fos*	GATGTTCTCGGGTTTCAACG	GGAGAAGGAGTCGGCTGG
m*Hsl*	GGCTTACTGGGCACAGATACCT	CTGAAGGCTCTGAGTTGCTCAA
m*Atgl*	CTTGAGCAGCTAGAACAATG	GGACACCTCAATAATGTTGGC
m*Tet1*	TGGAGACTAGGTTTGGCCAGAA	CCCCGTGAACACTATCTTCTCAAT
m*Tet2*	TCTCAGGAGTCACTGCATGTTTG	GCTCCGACTTCTCGATTGTCTT
m*Tet3*	ACCTGCGATTGTGTCGAACA	GTGAGTGTAATATGGGCCTTCATCT
m*Dnmt3a*	CATGAACAGGCCTTTGGCA	TCTTGCAGCTCCAGCTTATC
m*Dnmt3b*	CCAAAAGGAGGCCCATTAGAG	GTACCCCGTTGCAATTCCAT
